# Role of Primary Care Physicians in management of Schizophrenia in Low and Middle Income Countries (LMIC): A systematic review

**DOI:** 10.12669/pjms.39.4.7286

**Published:** 2023

**Authors:** Muhammad Firaz Khan, Zia ul-Haq, Mian Mukhtar ul Haq, Adil Afridi, Saeed Farooq

**Affiliations:** 1 Muhammad Firaz Khan, FRC Psych. Department of Psychiatry, Lady Reading Hospital, Peshawar, Pakistan; 2 Zia ul-Haq, PhD, FCPS. Vice Chancellor, Khyber Medical University, Peshawar, Pakistan. Institute of Health & Wellbeing, University the of Glasgow, UK; 3 Mian Mukhtar ul Haq, FCPS. Department of Psychiatry, Lady Reading Hospital, Peshawar, Pakistan; 4 Adil Afridi, FCPS. Department of Psychiatry, Lady Reading Hospital, Peshawar, Pakistan; 5 Saeed Farooq PHD, FCPS. Faculty of Medicine & Health Sciences, Keele University, Honorary Consultant Psychiatrist, Midlands Partnership, NHS Foundation Trust, UK

**Keywords:** Schizophrenia, Psychosis, Primary care Physicians, Low and middle income countries

## Abstract

Many people with Schizophrenia lack the resources and access to mental health services especially in low and middle income countries. Integration of mental health into primary care services can be a cost effective way of reducing the disability associated with Schizophrenia. Our aim was to review the studies conducted on role of Primary care physicians in management of Schizophrenia in low and middle income countries. PRISMA guidelines were followed and we registered the study protocol at PROSPERO. Four Electronic Databases (Medline, Psycinfo, CINAHL and Embase) were searched in May 2022. Relevant articles after search were 504 of which 61 full text were examined. A total of 20 studies were included in the final review comprising of observational, experimental and qualitative studies.

Most studies reported on abilities of Primary care physicians including their knowledge, perceptions, skills and competencies in identifying and management of Schizophrenia and related Psychosis. Findings suggest that there is considerable amount of stigma, lack of awareness and social support about people diagnosed with Schizophrenia. Significant improvement was observed in diagnosis and management of schizophrenia by Primary care physicians who received appropriate training by experts in the field. This review suggests that appropriate training of General practitioners in diagnosing and treating schizophrenia can help in reduction of huge Treatment Gap in Schizophrenia. They can also be utilised in delivering psycho social interventions to improve overall quality of patient care.

## INTRODUCTION

Schizophrenia is a serious mental illness with distressing outcomes for a patient and family. It affects roughly 26 million people globally, and in terms of years lived with disability it is ranked fifth among men and sixth among women.^1^ Schizophrenia is responsible for nearly 1.3 percent of disability-adjusted life years worldwide and 1.6 and 0.8 per cent in the lower middle-income and low-income countries, respectively.^2^ Many people diagnosed with Schizophrenia lack access and resources to receive treatment especially in countries included in low and middle income category. This results in increased period of untreated psychosis, increased physical health problems, more relapses, and hospitalizations.

People diagnosed with schizophrenia have a considerable reduction in life expectancy, with an estimated 10-20 years of potential life time lost. A substantial proportion of excess mortality is attributable to physical illnesses, particularly those impacting cardiovascular, respiratory, and metabolic health. High stress, sedentary lifestyle, unhealthy diet and illicit drug use are the possible risk factors for these medical problems. Poor physical health can result from anti-psychotic medications as a result of metabolic syndrome i.e. Diabetes, Hypertension, hyperlipidemia and obesity.

Appropriate care is not received by majority of the patients with Schizophrenia.^1^ Almost Ninety percent of untreated Schizophrenia patients belong to Low- and Middle- income countries. Main issue for these people is lack of resources and access to mental health services. An extremely huge number of people suffering from psychiatric issues in LMIC do not get evidence-based treatments. This is the result of shortage of mental health professionals, services and financial support.^3^

There is substantial advantage of incorporating mental health services into primary care. Among the main benefits of integration is that the people would receive mental health care which they need. Probability of positive outcomes can further increase with the integration of services.^4^ There is well-documented neglect of mental health all over the world. In most countries and most of the times it happens at the level of health care policy making and planning by allocating inadequate resources to psychiatric services.^5^

Untreated Schizophrenia has a massive adverse financial and emotional impact on the patient and carers. Accessibility and use of mental health services are inadequate which has led to worrying treatment gap in schizophrenia. Disability associated with Schizophrenia can be reduced by integrating mental health services into primary care in developing countries. This can improve the quality of care and also be cost effective strategy.^6^ Primary care providers do not need to be experts in the diagnosis of Schizophrenia to provide effective care to these patients. This task can be attained by teamwork with a psychiatrist.

General practitioners should have basic knowledge of Schizophrenia, its aetiology, management, barriers and risks to this vulnerable population. This review aimed to find out the role of primary care physicians in management of Schizophrenia and related Psychosis in low and middle income countries. With this understanding, efforts can be made to teach and train primary care providers with clinically practical guidelines. This would help them to assess and manage these patients for provision of better quality of care in community. Our aim was to answer the questions below.

- What is the level of knowledge, attitude and skills of Primary Care Physicians (PCPs) in diagnosing and managing Schizophrenia?
- How able are PCPs in recognition of people with first episode psychosis and Schizophrenia?
- How experienced are PCPs in assessment of physical health conditions as per guidelines and recommendations (i.e. appropriate monitoring, examinations, and referrals)?
- Can PCPs assess and monitor Adherence with medication?
- Are PCPs trained in providing support to the family of people with Schizophrenia?

## METHODS

A protocol with key objectives and methods was developed and registered at the PROSPERO (International Prospective Register of Systematic Reviews) https://www.crd.york.ac.uk/prospero/display_record.php?ID=CRD42020173185

### Search strategy:

We searched the following electronic databases i.e. PsycInfo, CINAHL, Medline and Embase. Schizophrenia is labelled as a “serious mental illness” because it has a chronic course and adverse social outcomes. There is low participation of primary care physicians in its management especially in Low and middle income countries. The search was conducted from April to May 2022. We did not expect many experimental studies so it was decided to involve all types and designs of studies including qualitative, observational and experimental to address the review question. These included intervention studies both randomized controlled and quasi-experimental designs.

We also included all observational study designs e.g. Cross-sectional, Cohort, and Case-control studies that address any aspect of research questions lined above. Following search terms were used: (Schizophrenia and other spectrum disorders OR Psychosis OR schizoaffective OR delusional disorder) (primary care physician*” OR “general practitioner*” OR “primary care doctor*” OR “family doctor*” OR “family physician*” OR “family practice” OR “general practice” OR “community practice” OR “community practitioner*” OR “community physician*” OR “community care Primary care Physicians) (“low and middle income countries” OR “low- and middle-income countries” OR LMIC OR India* OR Africa* OR Asia* OR “south America*” OR Balkan* OR “middle-east”) ((“quantitative stud*” OR “random* controlled trial*” OR “controlled trial*” OR “cross-sectional stud*” OR “longitudinal stud*” OR “cohort stud*” OR “case control stud*”).

Studies about People diagnosed with Schizophrenia or Schizoaffective disorder or related Psychosis living in the community and routinely managed by primary care physicians were included. For intervention studies we aimed to include any studies exploring pharmacological or psychological/psychosocial interventions for Schizophrenia administered or managed by primary care physicians. The region of patient setting was restricted to Low and Middle-income counties as per World Bank ranking.

Data extraction: Articles were screened based on the title, then abstract, and then full-text as to whether they meet eligibility criteria. Two researchers MFK and AA performed independent study selection and data extraction; differences were decided by agreement or a third researcher. Data was extracted into table, regarding study characteristics i.e. year published, location, study design, and sample size and variables related to main outcomes.

Two reviewers assessed the quality of the studies independently by using Risk of Bias tool developed by Cochrane for randomised trials.^7^ Quantitative data was first evaluated in order to identify patterns. Afterwards different interventions and their settings were examined if they were comparable, to inform later analysis. The studies identified were heterogeneous therefore narrative synthesis was utilised by text to summarize the findings of multiple studies.

## RESULTS

The primary electronic searches showed 504 relevant abstracts and titles. After removal of duplication and screening of titles and abstracts, 61 were left and their full texts were examined. Finally, we included 21 papers in the review.

### Characteristics of included studies:

Twenty studies met the inclusion criteria and all of them are from low and middle income countries. These were conducted in Iran (5), Pakistan (3), Nigeria (3), India (2), Tunisia (2), Rwanda (1), Armenia (1), Suriname (1), Peru (1), Dominican Republic (1) and Nepal (1). Of the included studies, 12 were observational, three had qualitative design and 4 had experimental design. One study tested a diagnostic tool called CSP (clinical schedule for primary care psychiatry) to identify and diagnose different psychiatric conditions including Schizophrenia.

The total number of Primary Care Physicians which participated in these included studies was 978 while total number of patients involved in these studies were 16,107 (one study had 13514 patients over 10 years). Other healthcare professionals in these studies were 767 including medical students and post graduate trainees. We described the salient findings from each study because statistical analysis of these studies was not conducted due to heterogeneity.

A qualitative study among medical students and primary care physicians observed that Primary care physicians performed better than the medical students in socializing with people who had mental illness including Schizophrenia.^8^ Another study examined the perspectives and attitude of health professionals including General Physicians towards mental illness, mainly Schizophrenia concluded that health professionals had stigma about mental illnesses which was dependent on their level of contact with these patients.^9^ Similarly a study in Suriname determined that lack of awareness and social support, stigma and traditional medicine were the likely factors for lengthy duration of untreated psychosis.^10^

A diagnostic study was conducted to examine the consistency of a diagnostic tool called CSP (clinical schedule for primary care psychiatry) to recognise and diagnose psychiatric problems including Schizophrenia in community by Primary care physicians and later examined by Psychiatrist on ICD-10 criteria. Sensitivity of CSP was found to be 91% while specificity was 68% and found encouraging results.^11^

An experimental trial in Iran compared the efficiency of a home based aftercare intervention with usual treatment in people with Schizophrenia discharged from hospital. The intervention comprised of monthly visits from general practitioner and social worker to educate the patient and family. One year follow up showed significant improvement in intervention group in terms of re hospitalization, illness severity and psychotic symptoms compared to the control group.^12^ Another Randomized controlled trial compared performance of General practitioners applying collaborative care program CC and the GPs in control group. Performance of primary care physicians was evaluated regarding their skills in history taking, communication, treatment plan and prescription. Physicians in collaborative care group performed considerably better in making therapeutic relationship and management of psychosis than the GPs in control group.^13^

A pilot cluster RCT performed in Nepal examined the effectiveness of an intervention to decrease stigma amongst primary healthcare practitioners including GPs in collaboration with patients who lived with Psychosis and showed positive results.^14^ A before and after study (quasi-experimental) was conducted in Armenia to examine effectiveness of brief training intervention (interactive workshops and face to face discussions of cases) on Depression and Psychosis among GPs and nurses. It resulted in significant improvement in the knowledge, attitude and practice of the primary care physicians.^15^

An observational study conducted in Pakistan aimed to measure the understanding and practices of primary care physicians about identification and management of schizophrenia. Out of 114 GPs, only 14(10.3%) practitioners had satisfactory understanding about management of schizophrenia, while 100(87.7%) had poor or no knowledge.^16^ Another study showed that 62% of General Practitioners self-perceived themselves as capable in appropriate diagnosis of schizophrenia (62.0%). Regarding pharmacological treatment, 37.6% observed themselves to have the skills in managing schizophrenia.^17^ For example Abiodun et al explored the skill of Primary Care Physicians to recognize children with psychiatric problems including schizophrenia. The PCPs recognized 12 of the 157 children to have some form of mental disorder while K-SADS (Children version of schedule of Affective disorders and schizophrenia to make DSM 4 Diagnosis) identified 40(22.5%) of 157 as having psychiatric disorder. This showed that PCPs had less ability in diagnosis as compared to K-SADS.^18^

Another study observed that Primary Care Physicians had better understanding about Depression and Psychosis than self-harm and substance abuse. Negative behavior was found about the dangerousness of people with psychiatric problems, and PCPs had little belief in their capabilities to manage psychosis.^19^ Another study showed that 34.3% of primary care physicians struggled in diagnosis of mental health problems while 48.6% had difficulty in management of psychotic patients.^20^ During a study, Mental Health Gap Action Program (MhGAP) training for General Practitioners and other healthcare staff of District Bannu resulted in significant improvement in knowledge, identification and management of mental illnesses including psychosis.^21^ A Tunisian study compared the attitudes of family physicians and non-medical students and did not find any significant difference regarding social distancing and dangerousness.^22^

**Table-I:** Types of Studies with sample size and outcome

Study	Author	Year	Country	Study design	Sample size	Outcome
1	Zafra-tanaka et al^18^	2019	Peru	Cross sectional	434	Self-perceived competence in diagnosing and treating mental health disorders including Schizophrenia
2	Irfan et al^17^	2015	Pakistan	Cross sectional	135	Knowledge and practice skills in diagnosis and treatment of schizophrenia
3	Abiodun et al^19^	2011	Nigeria	Cross-sectional two-stage study	350	Ability to identify children with mental health problems including Psychosis
4	Ighodaro et al^9^	2014	Nigeria	Qualitative	91	Beliefs and Attitudes towards mental illnesses among Nigerian medical personal
5	Amini et al^14^	2016	Iran	RCT	52	Performance evaluation of Primary care physicians in treatment of people with Psychosis
6	Caplan et al^10^	2016	Dominican republic	Qualitative	37	Attitudes of healthcare providers towards mental illness including schizophrenia
7	Feten Fekih-Romdhane et al^23^	2022	Tunisia	Cross-sectional	186 family physicians	Comparison of attitudes between family physicians and non-medical students towards schizophrenia
8	Atousa van beek et al^11^	2022	Suriname	Qualitative	8 GPs	Factors related to duration of untreated psychosis (DUP)
9	Lara Mroueh et al^15^	2021	Armenia	Quasi-experimental	111 GPs	Improvement in knowledge, attitudes and practices of primary healthcare workers
10	Khadivi et al^26^	2011	Iran	Prospective uncontrolled study	13514	Efficiency of integrated mental health program into primary care
11	Barikar C. Malathesh et al^28^	2021	India	Cross sectional	22 GPs	Impact of technology driven training in identification of mental health problems
12	Mottaghipour et al^27^	2010	Iran	Descriptive	8	Usefulness of training health professionals in adherence to protocol of psycho-education
13	Imran, A., & Haider, I,.I^24^	2007	Pakistan	Cross sectional survey	434	Perceptions and behaviour of doctors and medical students towards psychiatry patients
14	A. Binitie^25^	1981	Nigeria	Observational	83	Burden of psychiatric morbidity in a rural population compared to urban population and differences in diagnosis by a GP and a psychiatrist
15	Spagnolo et al^20^	2018	Tunisia	Observational	112	Understanding and attitude of PCPs and skills to manage psychiatric issues
16	Bolhari et al^21^	2012	Iran	Cross sectional	1371 51 GPs	Evaluation of integration of mental health program into primary health care system
17	Sharifi et al^13^	2012	Iran	RCT	130	Improvement in Re- hospitalization rate, psychotic symptoms, service satisfaction and illness severity.
18	Brandon A. Kohrt et al^16^	2018	Nepal	Pilot cluster RCT	88 GPs	Feasibility of anti-stigma intervention during mental health training of GPs and accuracy of diagnosis of mental illnesses
19	Kulkarni et al^12^	2019	India	diagnostic	180	efficiency of clinical schedules for primary care psychiatry (CSP) in diagnosis of psychiatric disorders
20	Humayun et al^22^	2017	Pakistan	Pre and post training study		Identification of gaps in Knowledge and management of common mental disorders including psychosis

**Fig.1:**
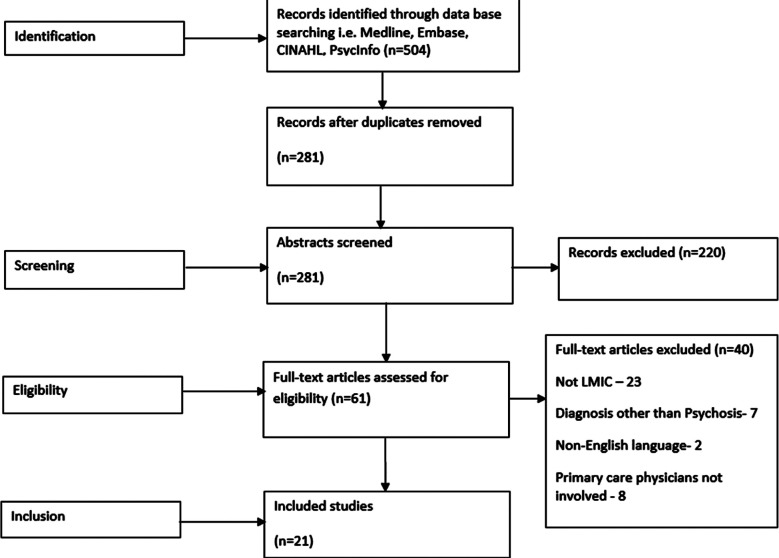
PRISMA diagram shows initial search process in selecting texts for final review. Total number of full text articles examined were 61 of which 21 studies were selected for final review. (Reasons for exclusion given).

Four studies mentioned to have used experimental designs to assess different interventions. Variable details were provided on Cochrane tool for Risk of Bias assessment of RCTs. One quasi-experimental study did not provide any details about quality criteria so we could not apply the Cochrane tool to this study.^8^ The other three studies’ assessment of quality is given in Fig.1. Only one study^18^ had a low risk of bias score on most items while others had medium to high risk of bias on Cochrane tool for the assessment of intervention studies.

## DISCUSSION

One of the main findings of this review is that there is dearth of literature on involvement of PCPs in treating schizophrenia in LMIC. We could identify only 21 studies after extensive literature search, most of which were of average quality and heterogeneous in nature. The studies showed that there is lacks of awareness about the involvement of PCPs in treatment of schizophrenia in LMIC. There is some evidence that effective psychosocial interventions delivered by primary care physicians can be useful in management of people with schizophrenia. There is need for improving training for PCPs as Irfan et al^16^ showed that almost half of the GPs had difficulty in diagnosing and treating Psychosis and related illnesses.

Brief training intervention for General Physicians can improve their level of knowledge, attitude and practice about schizophrenia. Overall perceptions and attitude of primary care physicians towards severe mental illness and these patients was better than medical students which means that dealing with these patients can reduce the stigma and apprehensions of these health professionals.^7^ Inclusion of Psychiatry as a major subject is essential for raising awareness about mental health among medical students as currently their views are negative due to lack of knowledge. Collaboration between GPs and specialist psychiatric services can be a useful strategy to further increase their understanding of these disorders.

These patients were perceived as dangerous which resulted in distress and less empathy among them. Primary care physicians felt they were not trained in managing mental illness and there was lack of support from families and community.^8^ Psycho education and nature of the illness can be better explained to these GPs with formal training in Schizophrenia and other Psychosis which is the job of Psychiatrists and health department. According to one study health professional including GPs had a higher degree of stigma towards people with severe mental illness.^23^ This needs to be addressed on priority basis as it can be a major barrier in integration of mental health services into primary care. Media, government and other organizations need to initiate campaigns and awareness programs for the all segments of population.

**Fig.2:**
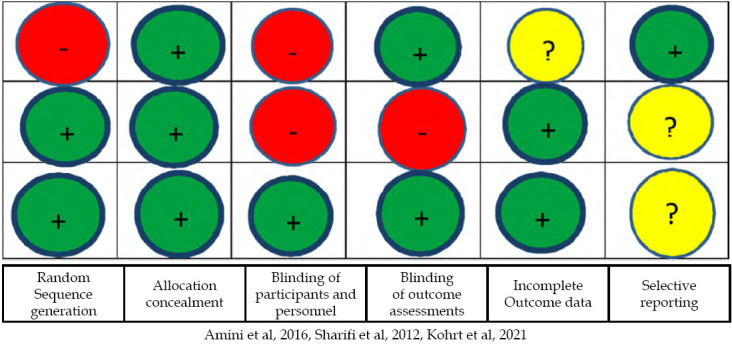
Risk of Bias assessment. (□ High risk of bias), (+ Low risk of bias), ( ? Not menti oned).

One study showed that Clinical Schedule for Primary care (CSP) can be a helpful tool in diagnosis of psychiatric illness in primary care.^9^ Other similar tools need to be developed for local populations which can be utilized by GPs in diagnosing severe mental illness. Patients were assessed by GPs and then re assessed by Psychiatrists which demonstrated 60% agreement on diagnosis of Psychosis.^24^ This can be further scaled up to other mental health problems as well. One study evaluated efficiency of trained General Practitioners (GPs) to screen, treat and follow up patients with psychiatric disorders including Psychosis.^25^ Health professionals including GPs were trained to deliver psycho education to the families of patients and then analysed in terms of adherence. The results were found to be satisfactory.^26^

Further studies are required to assess the time and skills needed to train GPs to deliver psychosocial interventions effectively. The understanding and training skills of general practitioners were found to be unsatisfactory regarding Schizophrenia diagnosis and treatment.^13^ There is dire need to integrate psychiatric disorders management with medical education to develop the necessary skills to deal with mental health disorders.^14^ Protocol or guideline and collaborative educational program to improve the child mental health knowledge among the PCPs can be a useful approach.^15^ Involvement in previous mental health training and psycho education were associated with better mental health competencies.

Mental health training may help further develop mental health skills in non-specialists, and incorporate mental health into community health care.^17^ Performance of GPs can be improved in identifying and management of Psychosis by interactive training and health information system. Their ability to identify patients with mental health problems can be assessed by using simulated patients. Collaborative care approach enabled them to make good therapeutic relationship with their patients.^11^ Hybrid training (online sessions plus onsite training) has been reported to be effective for GPs in identification of common and severe mental disorders and can be utilized as a task shifting tool to integrate psychiatric services in primary care.^27^

Duration after discharge from Hospital is a very important phase of management and regular follow up at home resulted in reduced re hospitalization and higher overall satisfaction of the patient but it did not improve quality of life.^11^ Aftercare at home can be a very useful approach for people with chronic mental illness especially patients with poor medication adherence but it can be difficult with limited resources.

There has been discussion around developing Community Psychiatry in Pakistan for last many years but practically there is not much significant in this regard especially for Psychosis. We can learn from a few trials in LMIC which examined GPs involvement in management of Psychosis but we need to develop interventions according to our very own settings to effectively utilize primary care physicians for this purpose.^12^-^14^ Recently a Cluster RCT (STOPS+) has been conducted in Peshawar, Pakistan to test effectiveness of a psychosocial intervention in people with Schizophrenia to improve medication adherence and functionality.^28^ Hopefully this will lead to more studies in future on this subject.

We did not find any studies on GPs role in providing support for the family of people with Psychosis so that aspect needs to be explored and developed in low and middle income countries. Besides this, monitoring of physical health of these patients is a major challenge and can be overcome by arranging training and resources for primary care physicians.

### Strengths:

We used a broad search approach to find all pertinent studies on the topic. Main strength of this review is that this is the first study of its kind which has searched to collect information in low and middle income countries about this particular topic. We kept inclusion criteria broad to examine all types of studies conducted. More than two researchers looked at all the studies to check for inclusion criteria.

### Limitations:

There were two RCTs and one Cluster RCT and they were heterogeneous due to which meta-analysis and quantitative assessment of interventions was not possible. Studies were of average quality and Risk of bias was not explained clearly. Many observational studies did not describe the participants and outcomes clearly and could not be combined to assess on STROBE checklist due to different characteristics. Only English language articles were included so there are chances that we may have missed related studies which were published in other languages. We found only three studies from Pakistan which were all observational so there is clearly a need for conducting more studies especially experimental trials to test different interventions in our own settings and culture.

## CONCLUSION

Primary care physicians can be utilized for early identification and management of Psychosis in community after receiving appropriate training. They can also be useful in teamwork with other mental health professionals to provide after care at home after discharge from hospital. Psychosocial and family interventions can also be delivered by GPs when appropriately trained. They can also be trained in crisis management for suicide and aggression among patients and can handle psychiatric emergencies to some degree. They need to be aware of their limitations and when to refer to specialist services. We need further studies to develop cost effective interventions for better patient care of Psychosis at primary health centers.

### Authors Contribution:

**MFK:** Conceived and designed & is responsible for integrity of research. **AA and MMH:** Did reviewing of studies and editing of manuscript. **SF and ZH:** Did review and final approval of manuscript.
